# Anti-proliferative and anti-migration effects of Polish propolis combined with *Hypericum perforatum* L. on glioblastoma multiforme cell line U87MG

**DOI:** 10.1186/s12906-016-1351-2

**Published:** 2016-09-20

**Authors:** Maria H. Borawska, Sylwia K. Naliwajko, Justyna Moskwa, Renata Markiewicz-Żukowska, Anna Puścion-Jakubik, Jolanta Soroczyńska

**Affiliations:** Department of Bromatology, Faculty of Pharmacy with Division of Laboratory Medicine, Medical University of Bialystok, Mickiewicza 2D, 15-222 Bialystok, Poland

**Keywords:** *Glioblastoma multiforme*, Propolis, *H. perforatum* L.

## Abstract

**Background:**

Propolis and *Hypericum perforatum* L. are natural products which contain many active compounds and have numerous beneficial effects, including an antitumor effect. *Gliobmastoma multiforme* (GBM) is a common primary brain tumor with poor prognosis and limited treatment options. In this study, the effect of propolis (EEP) combined with *H. perforatum* L. (HPE) on glioblastoma cell line U87MG was investigated for the first time.

**Methods:**

Anti-proliferative activity of EEP, HPE and their combination (EEP + HPE) was determined by a cytotoxicity test, DNA binding by [^3^H]-thymidine incorporation and cell migration assay. Anti-metastatic properties in U87MG treated with EEP, HPE and EEP + HPE were estimated on cells migration test (scratch assay) and metalloproteinases (MMP2 and MMP9) secretion (gelatin zymography).

**Results:**

Combination of HPE and EEP extracts was found to have a time- and dose-dependent inhibitory effect on the viability of U87MG cells. This effect was significantly higher (*p* < 0.05) when compared to these two extracts applied separately, which was confirmed by the significant reduction of DNA synthesis and significantly higher mitochondrial membrane permeabilization. A significant decreasing in migration cells and in pro-MMP9 and pro-MMP2 secretion in U87MG cells were demonstrated after exposure to combination of EEP (30 μg/ml) with HPE (6.25 μg/ml).

**Conclusions:**

In this study, the combination of ethanolic extract from propolis and ethanolic extract of fresh-cut *H. perforatum* L. was proved the ability to reduce invasiveness of glioma cells through the inhibition of MMP2 and MMP9 secretion and suppression of cell migration. It has a more potent anti-proliferative effect on U87MG glioma cell line compared to using propolis and *H. perforatum* L. separately. Further studies are required to verify whether the examined extracts can activate apoptotic pathways.

**Electronic supplementary material:**

The online version of this article (doi:10.1186/s12906-016-1351-2) contains supplementary material, which is available to authorized users.

## Background

*Glioblastoma multiforme* (GBM) is the most malignant human brain tumor, with median survival of one year. The malignant glioma cells are able to extensively invade surrounding brain parenchyma. The matrix metalloproteinases (MMPs) contribute to cancer cell invasion and metastasis through the cell-surface extracellular matrix (ECM) degradation [1]. Common resistance to chemotherapy and lack of effective therapy have encouraged search for new forms of treatment. It is common that oncological patients often use supplements with medicinal plant or bee products (such as propolis) to support the therapy [2, 3]. The use of various combinations of plant extracts may improve the effectiveness of treatment but, on the other hand, it can be dangerous because of their possible interactions [4].

*Hypericum perforatum* L. (commonly known as St. John’s wort) is best known for its use in treatment of depressive disorders, but its mechanism of action is unclear. Most researchers confirm that its effect is related to the return uptake of serotonin at the neural synapses and inhibition of monoamine oxidase (MAO). Currently, many phyto- and nutraceuticals, tinctures, teas, juices with St. John's Wort are available on the market [5]. St. John’s wort extract is also used in treatment of wound healing, bacterial and viral infections [6, 7]. The activity of *H. perforatum* L. is associated with its active compounds, like naphthodianthrone (hypericin, isohypericin), flavonoids (kaempherol, quercetin, rutin), prenylated phloroglucinols (hyperforin, adhyperforin), tannins, aromatic acids (caffeic, p-coumaric, chlorogenic) [8, 9]. Many studies have already confirmed the antitumor effect of *Hypericum perforatum* L. and hypericin (one of the major active components) [10–12]. Noell et al. [13] indicated the selective accumulation of hypericin in the tumor implantation (C6 glioma) in rats. Moreover, hypericin was shown to be a promising anticancer compound in the photodynamic therapy [14].

Propolis is a natural bee product characterized by the rich chemical composition, where the most active compounds are flavonoids (e.g. chrysin, apigenin, pinocembrin, pinobanksin, kaempferol), aromatic acids (e.g. *p-*coumaric, ferulic), esters (caffeic acid phenethyl ester – CAPE) [15, 16]. The antibacterial, antifungal, antiviral and anti-inflammatory activity were proved by many researchers [17–19]. The recent in vitro studies have also confirmed a wide anti-cancer effect on various cell lines such as human lung cancer (A549) [20], human prostate cancer (PC3) [21], human myeloid leukemia (U937) [22]. In our previous studies, we examined the influence of propolis extract and other bee products on human glioblastoma cells (U87MG) [23, 24]. Propolis presented the highest cytotoxic properties compared to other tested bee products and reduced the activity of NF-кB in the glioblastoma cell line (U87MG). Moreover, the ethanolic extract of propolis synergistically enhanced the activity of temozolomide against the proliferation of U87MG cells.

The aim of our study was to examine the effect of *H. perforatum* L. combined with propolis on U87MG glioblastoma cell line.

## Methods

### Reagents

Minimal essential medium eagle (MEM) with l-glutamine (292 mg/L), fetal bovine serum (FBS), trypsin-EDTA, penicillin, streptomycin were purchased from PAA Laboratories GmbH (Pasching, Austria); calcium-free phosphate buffered saline (PBS) was from Biomed (Lublin, Poland), methylthiazolyl diphenyl-tetrazolium bromide (MTT), dimethyl sulfoxide (DMSO) were obtained from Sigma-Aldrich (St. Louis, MO, USA). Acrylamide/bis-acrylamide solution, ammonium persulfate, bovine serum albumin, brilliant blue *R,* bromophenol blue, calcium chloride, glycine, glycerol, sodium dodecyl sulfate (SDS), N,N,N’N’-tetramethylethylenediamine (TEMED), trichloroacetic acid, triton X-100, trizma base were obtained from Sigma-Aldrich (St. Louis, MO, USA). Prestained protein standard from BioRad. The scintillation cocktail was purchased from PerkinElmer (Boston, MA) and methyl-^3^H thymidine from MP Biomedicals, Inc. (Irvine, USA). Solutions: 7 (200 μg/ml 5,5’,6,6’-tetrachloro-1,1’,3,3’-tetra-ethylbenzimidazolocarbocyanine iodide (JC-1) and solution 8 (1 μg/ml DAPI in PBS) to NucleoCounter assays were obtained from ChemoMetec, Denmark. All other chemicals were of ultrapure grade and obtained from Sigma-Aldrich (St. Louis, MO, USA).

### Materials and extraction

Bee-propolis and the plant material *H. perforatum* L. were collected in the region of Podlasie, north-eastern Poland in 2014 (propolis) and 2015 (*H. perforatum* L.). Voucher specimens were deposited in the Department of Bromatology in the Medical University of Bialystok (ZB/PP/2014/04; ZB/PL/2015/02).

Propolis was crushed and 20 g were extracted in a shaker with 80 g of 95 % ethanol for 6 h in a darkened place. The extract was filtered and concentrated to dryness in a rotary evaporator under reduced pressure and controlled temperature. To obtain a dry extract the sample was lyophilized. The dry ethanolic extract of propolis (EEP) was protected from light and kept frozen at −80 °C. The yield of the prepared extract (% w/w), in terms of the starting material, was 39.5.

Fresh aerial parts of *H. perforatum* L. was cut and 80 g were extracted using a maceration technique with 500 g ethanol 95 % for 6 h at room temperature in darkness. Extraction was repeated two times over 48 h. The yield (% w/w) was 9.4. The mixture was filtered, concentrated and lyophilized. The obtained ethanolic extract of fresh-cut *H. perforatum* L. (HPE) was kept in −80 °C for future assays.

### Cell culture

The studies were performed on human glioblastoma U87MG cell line obtained from the American Type Culture Collection (ATCC, Rockville, MD, USA). The study did not require the approval of the ethics committee. The cells were cultured in a humidified incubator at 37 °C and 5 % CO_2_ atmosphere in growth medium - MEM supplemented with 10 % FBS; 50 U/mL penicillin and 50 mg/mL streptomycin.

### Morphological analysis under light microscopy

For the morphological analysis, a U87MG cell line was seeded in 100-mm dishes at 2.2 × 10^6^ cells/ml. The cells were treated with HPE (6.25 μg/ml), EEP (30 μg/ml) and combination HPE with EEP for 24 h. Then the morphological changes were examined based on the visual assessment and recorded under a light microscope (Olympus CKX41).

### Cytotoxicity assay

Lyophilized HPE and EEP were dissolved separately in DMSO and growth medium at 1000 μg/ml concentration as a stock solution. Further dilutions were made in culture medium. HPE was used at different concentrations ranging from 1.56 to 100 μg/ml and EEP at 30 μg/ml. The concentration of EEP (30 μg/ml) was chosen, based on the previous data [23]. The final concentration of DMSO was less than 0.1 %.

U87MG cells were seeded at a density of 2 × 10^4^ cells per well of a 96-well plate and grown for 24 h. Cells were incubated with prepared different concentrations of HPE and EEP for 24, 48 and 72 h. The control included 0.1 % DMSO. A 3-(4,5-dimethylthiazol-2-yl)-2,5-diphenyltetrazoliumbromide (MTT) viability assay was performed as previously described [24]. The absorbance at 570 nm proportional to the number of living cells was measured on a Multimode Plate Reader Victor X3 (PerkinElmer, Singapore). The data were expressed as a percentage of control. The cells from passage 8 to 11were used. The experiment was performed in triplicate and repeated at least three times independently.

### [^3^H]-thymidine incorporation

U87MG cells were seeded in 24-well plates at 1.5 × 10^5^ per well and grown for 24 h. Next, U87MG cells were treated with HPE (6.25 μg/ml), EEP (30 μg/ml), their combination and control (0.1 % DMSO). After 44- h incubation 0.5 μCi of [^3^H]-thymidine was added to each well. Cells were incubated for 4 h. Next the medium was removed and cells were washed twice with cold (4 °C) 0.05 M Tris-HCl and 5 % trichloroacetic acid. At the end cells were collected and placed into the scintillation cocktail. The level of [^3^H]-thymidine incorporated in the newly synthesized DNA strand in relation to cells proliferating during the S phase of the cell cycle was assessed by a scintillation counter. The cells from passage 11 to 13 were used.

### Mitochondrial potential assay

Mitochondrial membrane permeabilization was analyzed, applying the mitochondrial potential JC-1 stain assay with the NucleoCounter NC-3000 instrument and following instructions from the producer. Staining solutions used for the assay were the following: Solution 7 containing 200 μg/ml of 5,5’,6,6’-tetrachloro-1,1’,3,3’-tetra-ethylbenzimidazolocarbocyanine iodide (JC-1) and Solution 8 containing 1 μg/ml DAPI in PBS.

U87MG cells were seeded in 6-well plates at 0.7 × 10^6^ per well and grown for 24 h. Next, U87MG cells were treated with HPE (6.25 μg/ml), EEP (30 μg/ml), EEP with HPE and control (0.1 % DMSO). After 24-h incubation the mitochondrial potential was measured according to the NucleoCounter NC-3000 protocol. The cells from passage 14 to 16 were used.

### Migration assay (Scratch assay)

U87MG cells were cultured until more than 70 % of confluent growth for each well of 6-well plates, at 37 °C in a humidified atmosphere of 5 % CO_2_. Seeded cells in well plates were scratched with a 20–300 μl micropipette tip to the same length and width. U87MG cells were treated with HPE (6.25 μg/ml), EEP (30 μg/ml), EEP with HPE and control (0.1 % DMSO) and then incubated for 48 h. The images of each treatment group were captured at 100× magnification, using an Olympus CKX 41 microscope and KcJunior programme at each time point (0, 12, 48 h).

### Gelatin zymography

The gelatin zymography was used to assess the extent of pro-MMP2 and pro-MMP9 (as a precursors of MMPs: MMP2 and MMP9) activity [25]. The serum-free media with the study substances from subconfluent cells (U87MG) grown for 24 h in 100-mm plates were collected and concentrated 35-fold by ultrafiltration using 3 kDa concentrators and mixed with Laemmli sample buffer. After normalizing with the sample of the total protein aliquots of the samples were subjected to SDS-PAGE in a 10 % gel impregnated with 0.1 mg/mL gelatin. After electrophoresis, the gels were incubated in 2 % Triton X-100 for 30 min at 37 °C to remove SDS and in a substrate buffer (50 mM Tris–HCl buffer, pH 7.8, containing 5 mM CaCl_2_) for 20 h at 37 °C. Then, the gels were stained with Coomassie briliant blue R250. Gelatinolytic activity was detected as unstained bands on a blue background. The position of bands was identified on the basis of the molecular weight protein standard (BioRad). The cells from passage 7, 9 and 11 were used.

### Statistical analysis

All data were analyzed using Statistica software (version 12). The results were shown as a mean percent value of control ± standard deviation (SD) and are calculated from at least three independent experiments performed in triplicate; Significant differences calculated by means of Shaphiro-Wilk and Student-t tests. *P* values <0.05 were accepted as statistically significant.

## Results

### Morphological changes

**Fig. 1:**
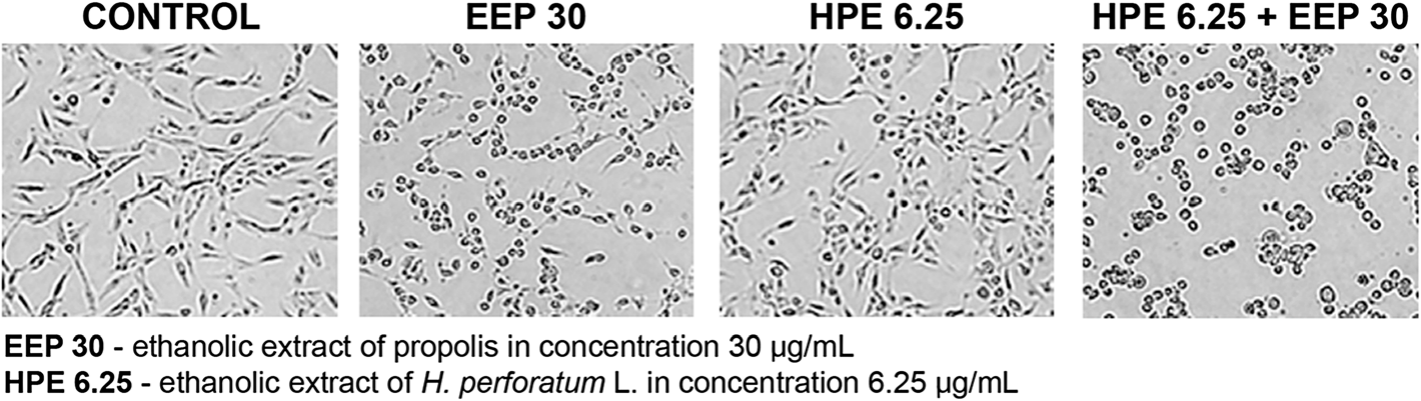
Morphological changes in U87MG cells after treatment with EEP, HPE and their combination. Legend: The effect of ethanolic extract of propolis (EEP - 30 μg/mL), ethanolic extract of fresh-cut *Hypericum perforatum* L. (HPE - 6.25 μg/mL) and their combining on morphology of U87MG cell line after 24 h treatment

In U87MG cell line, control cells showed a different branchy and polygonal shape, which is considered as the normal cell growth effect. The most visible changes in cell morphology were observed after treatment with EEP and its combination with HPE. The cells were rounded off, shrunk down and showed a decrease in their number (Fig. 1).

### Cytotoxicity effect of EEP, HPE and their combination

**Fig. 2:**
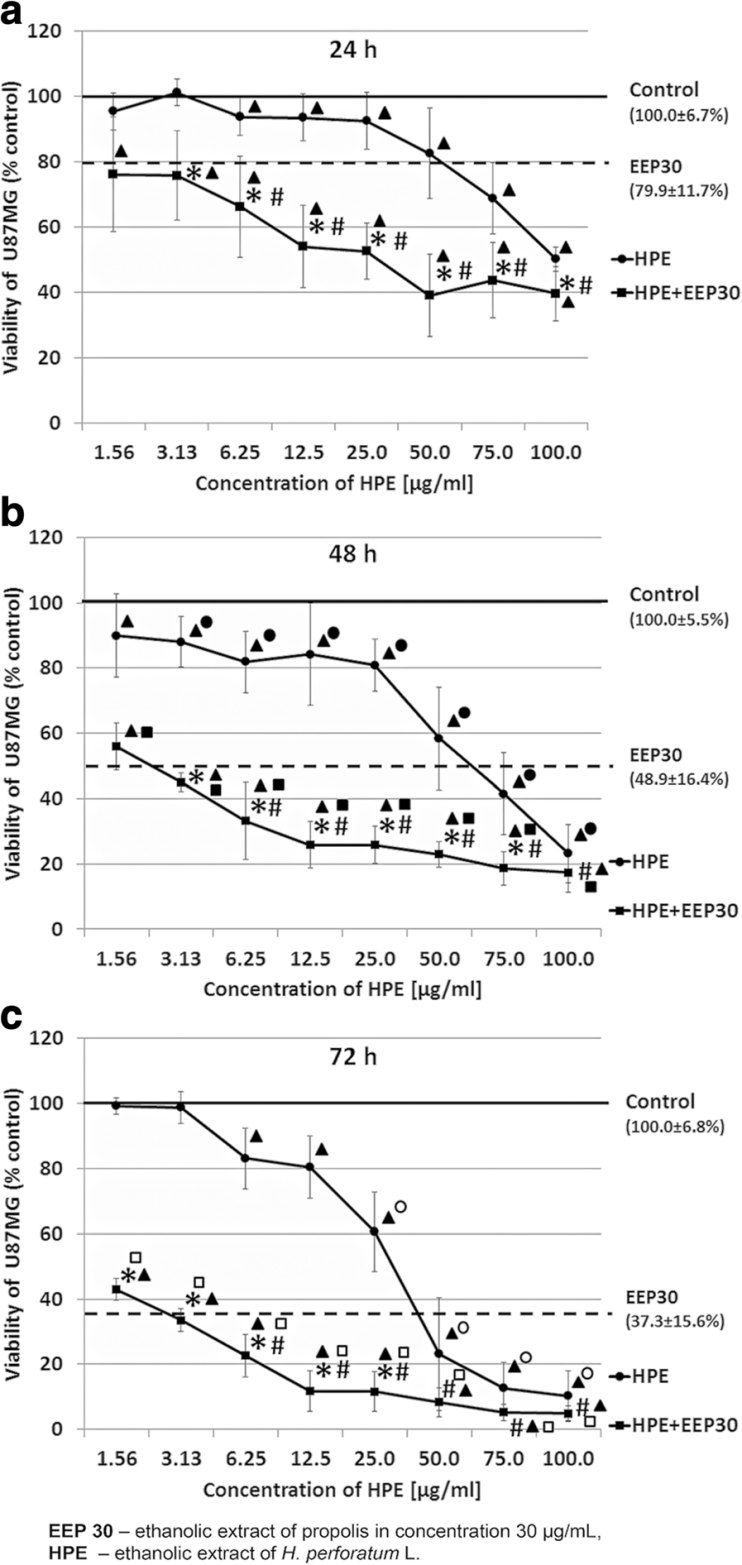
Viability of U87MG cells after treatment with EEP, HPE and their combination. Legend: Cytotoxicity effect of EEP (30 μg/mL), HPE (in concentrations 1.56–100.0 μg/mL) and their combinations after 24 (**a**), 48 (**b**) and 72 (**c**) hours of incubation. The results are presented as a percentage of control. All statistical analyses were performed using Student-t test (*p* < 0.02): ^*^HPE vs HPE with EEP; ^#^EEP vs HPE with EEP; ● HPE 24 h vs HPE 48 h; ○ HPE 48 h vs HPE 72 h; ■ HPE with EEP 24 h vs HPE with EEP 48 h; □ HPE with EEP 48 h vs HPE with EEP 72 h

The treatment of U87MG cells with HPE alone showed a time and dose-dependent decrement in the cell viability (Fig. 2). The significant inhibitory effect (viability less than 60 % of control) was found in a dose 100 μg/ml after 24 h; 50 and 100 μg/ml after 48 h and 25–100 μg/ml after 72 h incubation. After 24, 48 and 72 h of incubation with EEP viability of U87MG cells decreased to 80.0 ± 11.7 %, 48.9 ± 16.4 % and 37.3 ± 15.6 %, respectively. The cytotoxic effect of combination of HPE and EEP extracts on U87MG cells was significantly higher (*p* < 0.02) when compared to these two extracts used separately: after 24 h in concentrations of HPE from 6.25 to 100.0 μg/ml, after 48 h in concentrations of HPE from 6.25 to 75.0 μg/ml and after 72 h in concentrations of HPE from 6.25 to 25.0 μg/ml. Comparing different periods of incubation, the cytotoxic effect of HPE was significantly higher (*p* < 0.05) after 48 h than after 24 h in the concentrations from 3.13 to 100 μg/ml, while after 72 h it was significantly higher than after 48 h in the concentrations from 25 to 100 μg/ml. The cytotoxic effect of combination of EEP and HPE extracts on U87MG cells in different times of incubation was significantly higher (*p* < 0.05) after 48 h than 24 h, and after 72 h than 48 h in all used concentrations.

### The influence of EEP, HPE and their combinations on DNA synthesis

**Fig. 3:**
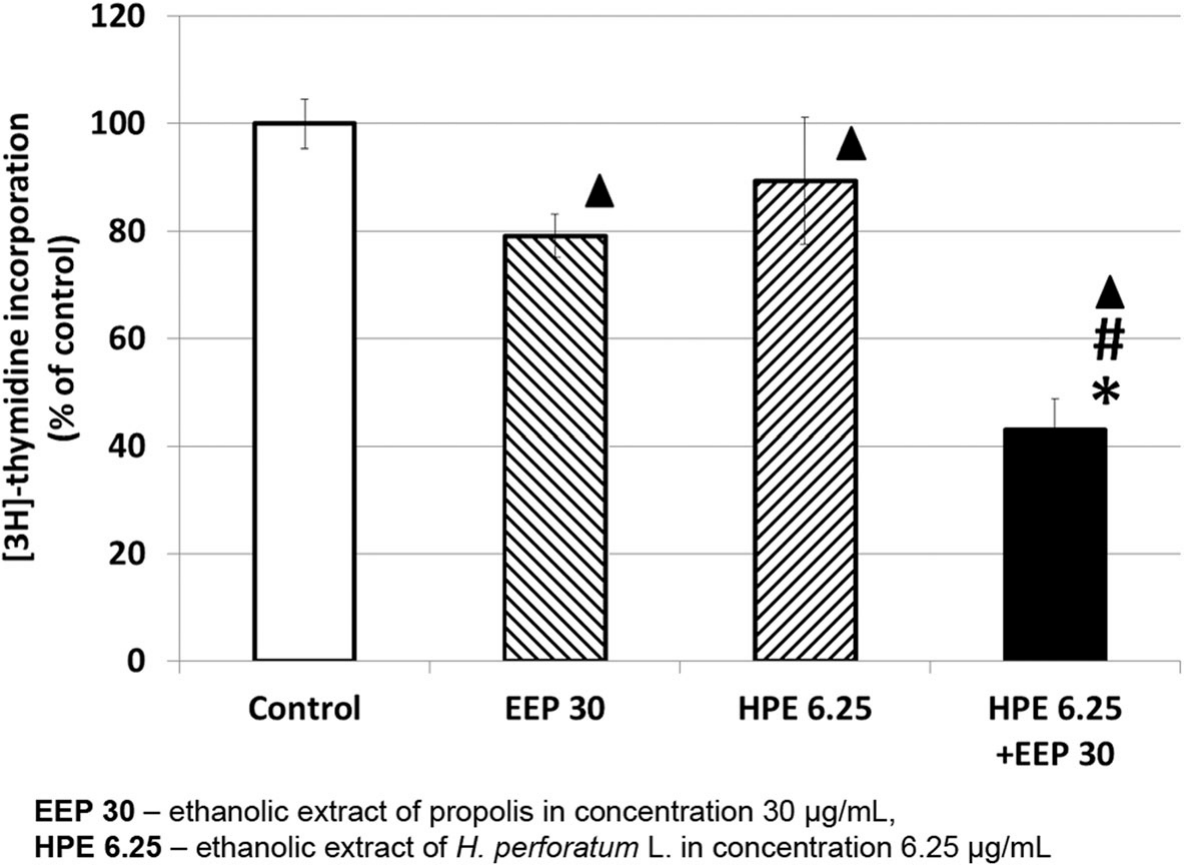
[^3^H]- thymidine incorporation into U87MG cells after treatment with EEP, HPE and EEP with HPE. Legend: ^3^H]- thymidine incorporation to U87MG cells after 48 h of incubation with EEP (30 μg/mL), HPE (6.25 μg/mL) and EEP (30 μg/mL) administered together with HPE (6.25 μg/mL). The results are presented as a percentage of control. Significant changes (*p* < 0.01): ^▲^EEP; HPE; EEP with HPE vs Control; ^*^HPE vs HPE with EEP; ^#^EEP vs HPE with EEP

Our study revealed a slight inhibitory potential on DNA synthesis: 79.2 ± 4.0 % and 89.4 ± 11.8 % of control, respectively, after 48 h of incubation in EEP (30 μg/ml) and HPE (6.25 μg/ml) used separately (Fig. 3). A statistically significant inhibitory effect was found for the combination of EEP (30 μg/ml) and HPE (6.25 μg/ml), where the reduction of DNA synthesis was 43.0 ± 5.8 % of control.

### Mitochondrial potential after treatment with EEP, HPE and their combinations

**Fig. 4:**
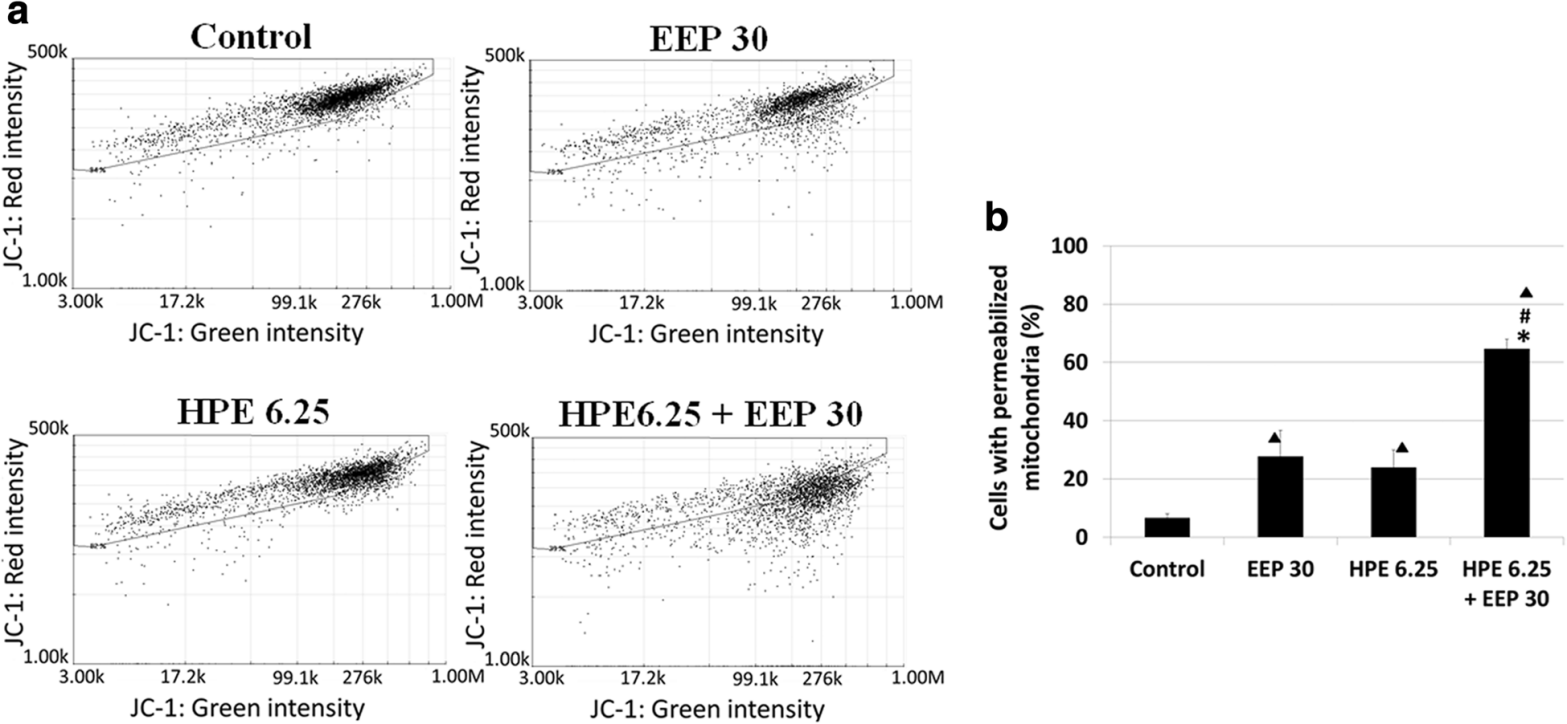
Mitochondrial membrane permeabilization in U87MG cells after treatment with EEP, HPE and their combination. Legend: Mitochondrial membrane permeabilization after treatment (24 h) with EEP (6.25 μg/mL), HPE (30 μg/mL) and combination of EEP (30 μg/mL) with HPE (6.25 μg/mL) in U87MG cells (**a**). Data is shown in percentage of permeabilized cells compared to control (**b**). Significant changes (*p* < 0.05): ^▲^EEP; HPE; EEP with HPE vs Control; ^*^HPE vs HPE with EEP; ^#^EEP vs HPE with EEP

The changes of mitochondrial membrane potential in U87MG cells after incubation with EEP (30 μg/ml), HPE (6.25 μg/ml) and combination of EEP with HPE were analyzed with the NucleoCounter NC-3000 instrument. The results obtained after treatment with combination of EEP (30 μg/ml) with HPE (6.25 μg/ml) demonstrated significantly higher (*p* < 0.05) permeabilization (64.7 ± 3.2 %) compared to the control (6.7 ± 1.5 %) as well as in comparison with EEP (27.7 ± 9.1 %) and HPE (24.0 ± 6.0 %) used alone (Fig. 4).

### The influence of EEP, HPE and their combinations on MMP2 and MMP9 secretion

**Fig. 5:**
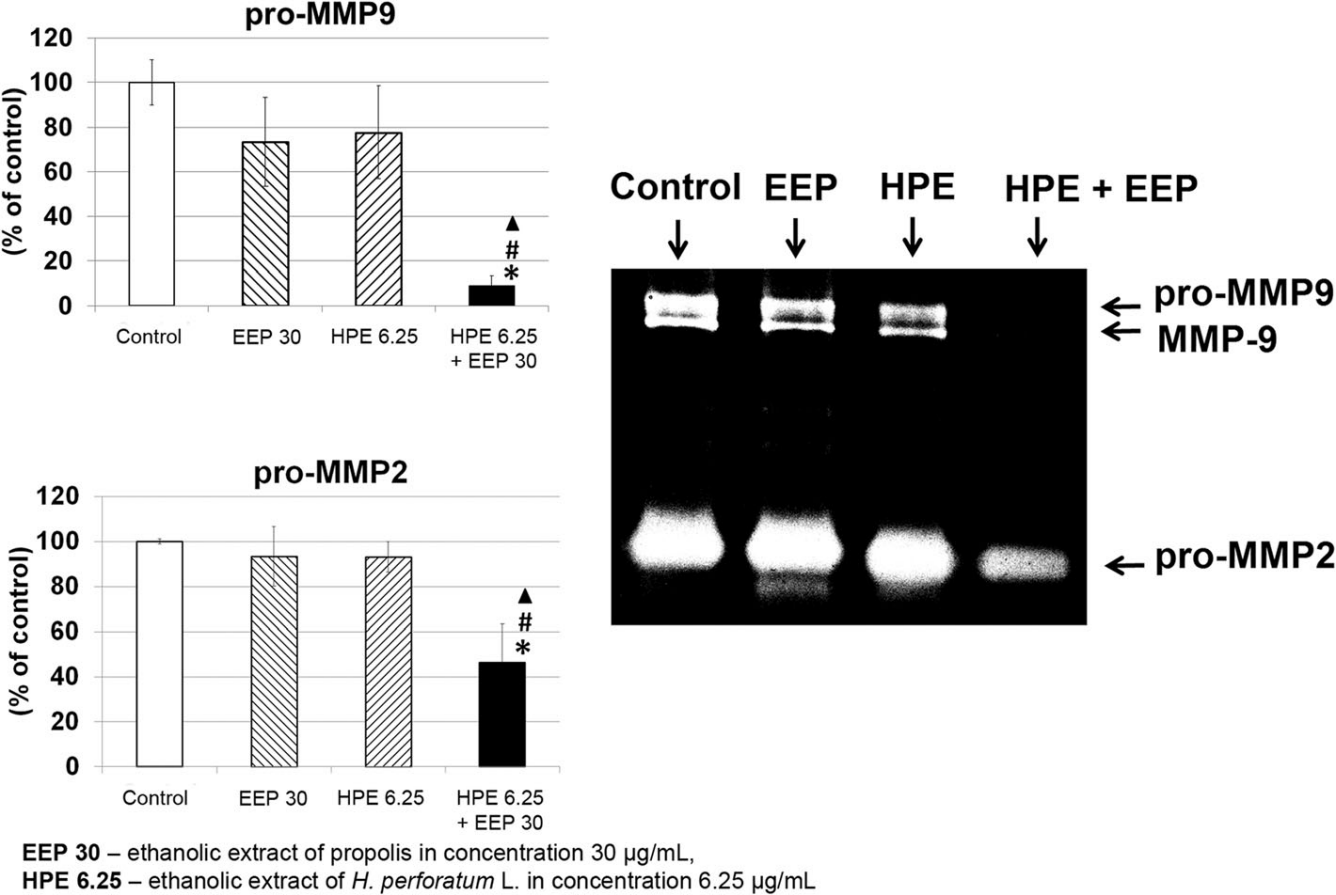
Inhibition of MMPs secretion from U87MG cells after incubation with EEP, HPE and their combination. Legend: Inhibition of MMPs secretion from U87MG cells after 24 h incubation with EEP (6.25 μg/mL), HPE (30 μg/mL) and combination of EEP (30 μg/mL) with HPE (6.25 μg/mL) (% of control). The picture is a representative gels of three independent experiments (medium concentrated by 35-fold; 7 μg of protein). Significant changes (*p* < 0.05): ^▲^EEP with HPE vs Control; ^*^HPE vs HPE with EEP; ^#^EEP vs HPE with EEP

No significant effect of EEP (30 μg/ml) and HPE (6.25 μg/ml) was revealed on the secretion of pro-MMP9 and pro-MMP2 (Fig. 5). A significant decrease (*p* < 0.05) in pro-MMP9 (8.7 ± 3.7 % of control) and pro-MMP2 (46.4 ± 16.9 % of control) secretion in U87MG cells was observed after exposure to combination of EEP (30 μg/ml) with HPE (6.25 μg/ml).

### Cell migration after treatment with EEP, HPE and their combinations

**Fig. 6:**
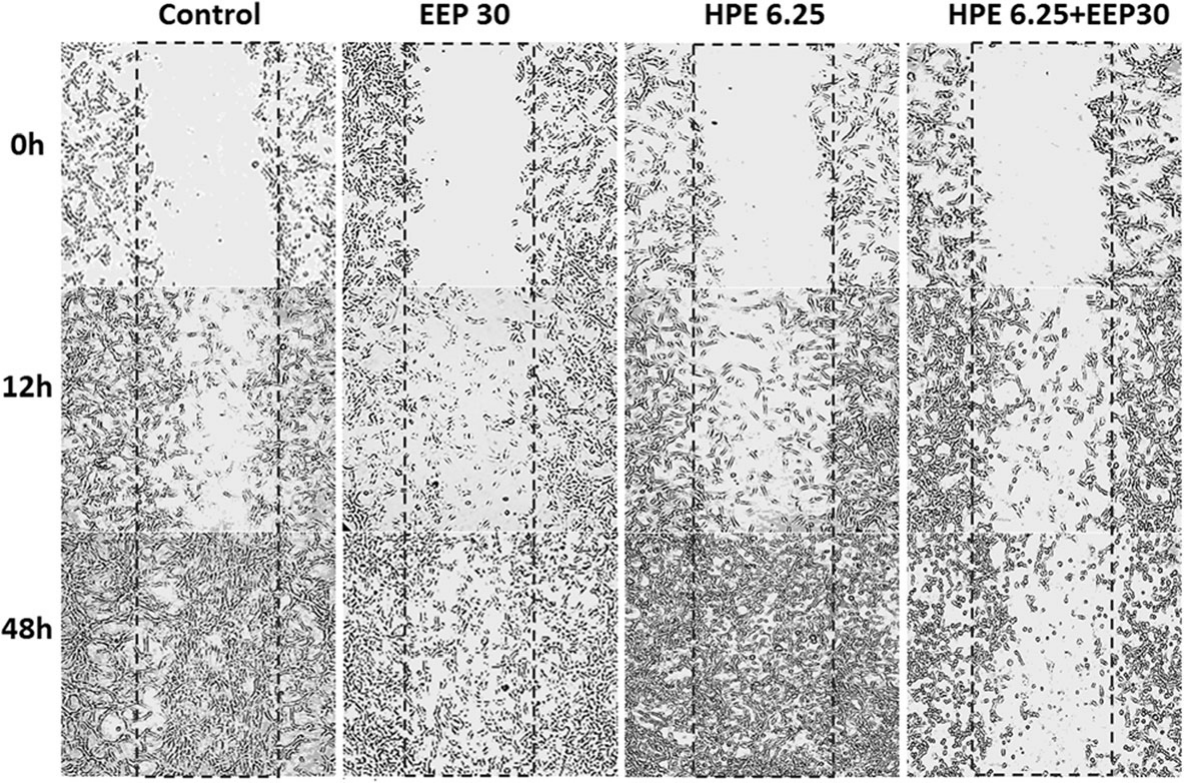
The effects of EEP (6.25 μg/mL), HPE (30 μg/mL) and combination of EEP (30 μg/mL) with HPE (6.25 μg/mL) on U87MG cells migration after 0, 12, 48 h of incubation

The effects of EEP (30 μg/ml), HPE (6.25 μg/ml) and combination of EEP with HPE on cells migration were evaluated using a scratch-wound assay and the results were compared to those from cells treated with 0.1 % DMSO as a control. As shown in Fig. 6, the combination of EEP with HPE decreased the migration of U87MG cells after 12 h compared to the control. More open spaces were observed after 48 h of treatment with combination of EEP with HPE. Moreover, the inhibition of cell migration by combination of EEP with HPE was more effective than that of EEP and HPE used alone.

## Discussion

The high mortality of patients suffering from glioma, difficulties in treatment, numerous side effects and failure of conventional therapy have resulted in search for new and more efficient substances, also of the natural origin, as potential anticancer agents. Thus, in this study, the effect of extract of propolis combined with extract of *H. perforatum* L on U87MG glioblastoma cells was examined via evaluating its anti-proliferative activity.

*H. perforatum* L. is best known for its use in treatment of mild to moderate depressive disorders. Its mechanism of action is unclear. Most researchers confirm that its effect is related to the return uptake of serotonin at the neural synapses and the inhibition of monoamine oxidase (MAO). The most active ingredients of *H. perforatum* L. are hypericin and hyperforin. The highest amount of hypericin (0.115 %) is obtained after ethanolic extraction, while the best efficiency for hyperforin (2.397 %) is obtained after supercritical carbon dioxide extraction [26]. Hypericin is the most widely examined ingredient of *H. perforatum* L. Noell et al. [13] confirmed for the first time that hypericin accumulated selectively in the implantation of a tumor (C6 glioma) in rats. The authors suggested that hypericin could be an effective fluorescence marker for detection of this type of a tumor. Pfaffel-Schubart et al. [11] showed that the photosensitizer hypericin strongly inhibited the proliferation of U373MG glioma cells and induced apoptotic cell death. It is worth emphasizing that a number of different compounds present in the *H. perforatum* L. extract may interact with individual cell growth mechanisms, showing more evident effects than those used with each single agent. Roscetti et al. [10] showed that extract of *H. perforatum* L. had a significant dose- and time-dependent inhibition of K562 erythroleukemic cell growth and induced apoptosis, while the purified hypericin had a slight cytotoxic effect on the cell growth and no effect on the induction of apoptotic cell death. In another study the extract of *H. perforatum* L. showed the phototoxicity effect on human melanoma A375 cancer cells, where IC_50_ was observed in 78 μg/ml concentration [27].

In this study, HPE was found to have a time- and dose-dependent cytotoxic effect on glioblastoma cells, where this significant inhibitory effect on viability (less than 60 % of control) was determined in a dose of 50 μg/ml after 48 h of incubation.

Propolis – a natural bee product has been the subject of many studies due to its anticancer properties. Borges et al. [28] showed a strong inhibitory effect on the cell growth on glioblastoma (U251 and U343) and fibroblast cell lines (MRC5) after treatment with Tubi-bee propolis. Propolis from Croatia and Brazil showed an antitumor effect on mammary carcinoma cells (MCA), human epithelial carcinoma cell line (HeLa) and Chinese hamster lung fibroblast cells (V79) [29]. In our study, it was demonstrated that EEP in the concentration of 30 μg/ml significantly inhibited the growth in U87MG glioma cells after 24, 48 and 72 h of incubation. Our previous study revealed the similar effect using propolis obtained from Poland [23]. The main goal of our research was to check whether the combination of EEP and HPE would enhance the antitumor effect or on the contrary - cause negative interactions and a reverse effect. Our data showed that the inhibition of U87MG cells growth after incubation with combination of EEP and HPE was significantly higher when compared to these two extracts separately. To the best of our knowledge, this study is the first to show the cytotoxic effects of *H. perforatum* L. combined with propolis on glioblastoma cell line.

In order to confirm that this decreased cells’ viability was caused by weakening their ability to proliferate, the EEP and HPE impact on DNA biosynthesis was examined in a [^3^H]-thymidine incorporation assay. In our study, it was determined that the highest inhibition of proliferation occurred after treatment with combination of EEP and HPE when compared to these two extracts applied separately. Aso et al. [30] found that Turkish propolis showed a potent antitumor activity reflected in DNA, RNA inhibition and protein synthesis. We observed disruption of the mitochondrial membrane potential, which may indicate apoptosis or necrosis in cells treated with EEP and HPE together [31]. Further studies are required to verify whether EEP with HPE can activate apoptotic pathways.

The metastasis process of cancer is strongly connected with proteolytic enzymes – metalloproteinases (MMPs) degrading extracellular matrix. MMPs, especially, MMP9 and MMP2 are overexpressed during glioma development [32]. Our study results showed that the secretion of pro-MMP9 and pro-MMP2 from U87MG cells was significantly decreased after treatment with EEP and HPE together compared to the control. The significantly stronger inhibition was observed in comparison with EEP or HPE used separately. Our previous study with other bee products proved that beebread and various honeys also inhibited the secretion of MMP9 and MMP2 in glioblastoma cells [24, 33]. An integral part of metastasis that is required at virtually every step of the metastatic cascade is cell migration [34]. In our study, inhibition of U87MG cells migration was observed after treatment with combination of EEP and HPE. These results confirmed the ability to reduce invasiveness of glioma cells anti-migration potential of EEP and HPE combination.

Interestingly, the scientific research on the properties of active substances of *H. perforatum* L. has reported adverse drug interactions with other drugs that are metabolized by CYP3A4 due to hyperforin content, which induces CYP3A4 in the gut and liver [35]. Schreiber et al. [36] described the case of 63 year old woman with GBM, undergoing contaminant radiochemotherapy with temozolomide and a slight depressive episode during which she took *H. perforatum* L. Five months after cessation of fractionated radiation and adjuvant chemotherapy with temozolomide the patient developed bilateral amaurosis due to radiation-induced optic neuropathy (RION). The authors suggested that occurrence of RION may have been the result of radiosensitization by temozolomide, which could have been strengthened by hypericin.

## Conclusions

In this study, the combination of ethanolic extract from propolis and ethanolic extract of fresh-cut *H. perforatum* L. was proved the ability to reduce invasiveness of glioma cells through the inhibition of MMP2 and MMP9 secretion and suppression of cell migration. It has a more potent anti-proliferative effect on U87MG glioma cell line compared to using propolis and *H. perforatum* L. separately. Further studies are required to verify whether the examined extracts can activate apoptotic pathways.

## Additional file

Additional file 1: The row data of MTT, [^3^H]-thymidine incorporation and MMPs secretion tests. (XLSX 43 kb)

## Abbreviations

CYP3A4: Cytochrome 3A4
DMSO: Dimethyl sulfoxide
EEP: Ethanolic extracts of propolis
FBS: Fetal bovine serum
GBM: *Glioblastoma multiforme*
HPE: Ethanolic extracts of *Hypericum perforatum* L
JC-1: 5,5’,6,6’-tetrachloro-1,1’,3,3’-tetra-ethylbenzimidazolocarbocyanine iodide
MAO: Monoamine oxidase
MEM: Minimal essential medium eagle
MMP2: Metalloproteinase 2
MMP9: Metalloproteinase 9
MMPs: Metaloproteinases
MTT: Methylthiazolyl diphenyl-tetrazolium bromide
PBS: Phosphate buffered saline
pro-MMP2: Pro-metalloproteinase 2
pro-MMP9: Pro-metalloproteinase 9
RION: Radiation-induced optic neuropathy
SD: Standard deviation
TEMED: N, N, N’N’-tetramethylethylenediamine
U87MG: Human *glioblastoma multiforme* cell line
